# Nutritional Support Best Practices in Pediatric Blood and Marrow Transplant Patients: An Integrative Review

**DOI:** 10.3390/children11060637

**Published:** 2024-05-25

**Authors:** Jessica D. Murphy, Heather J. Symons, Kenneth R. Cooke

**Affiliations:** 1 School of Nursing, Johns Hopkins University, Baltimore, MD 21287, USA; jmurph89@jhu.edu; 2 Sidney Kimmel Comprehensive Cancer Center, Johns Hopkins University School of Medicine, Baltimore, MD 21287, USA; hsymons2@jhmi.edu

**Keywords:** malnutrition, pediatrics, oncology, BMT, HSCT, stem cell, transplant, cellular therapy, nutrition

## Abstract

Nutrition is vital to the long-term survival of children undergoing blood and marrow transplantation (BMT), but there is no standardization on how to optimize the nutritional status of these patients. A literature search was performed to evaluate nutritional support approaches currently in practice for pediatric patients who are undergoing BMT. CINAHL, Embase, and Cochrane databases were searched for peer-reviewed articles evaluating nutritional interventions for BMT recipients aged 20 or younger. Nine articles published between 2019 and 2023 were found and reviewed. The nutritional support varied between enteral nutrition, parenteral nutrition, a combination of both, and prophylactic feeding tube placement. The post-transplant outcomes identified as associated with alterations in nutritional regimens included length of stay, date of platelet engraftment, incidence of acute graft-versus-host disease, infection rate, and overall survival. The use of any amount of enteral nutrition compared to parenteral alone was beneficial. Complications during BMT can potentially be decreased via prioritizing enteral nutrition over parenteral. The paucity of literature on this topic highlights an unmet need in the field. Future research should focus on ways to optimize the nutritional support of transplant recipients, including increasing the likelihood of enteral feeding tube placement and utilization.

## 1. Introduction

Malnutrition, or undernutrition, is an imbalance between the required nutrient intake and the actual intake, leading to deficiencies in caloric energy, protein, and micronutrients [1]. Chronic disease in children contributes to malabsorption and increased metabolic demands [2], which, in turn, make many children, adolescents, and young adults with cancer or disorders of the blood and immune systems particularly vulnerable to malnutrition [3]. These patients often receive prolonged and intensive therapy, which impacts their optimal nutritional status and overall health [4]. The malnutrition rates in children with malignancies treated in developed countries vary widely: various articles report rates of 0–20% in leukemia patients, 0–31% in solid tumor patients, and up to 50% in high-risk neuroblastoma patients [5,6,7,8,9,10,11]. Malnutrition is also seen in children with transfusion-dependent anemias [12] and primary immunodeficiencies [13].

The BMT procedures are curative for many of these malignant and non-malignant conditions, including hemoglobinopathies, bone marrow failure syndromes, immunodeficiencies, immune dysregulatory syndromes, and metabolic disorders [14]. A preparative or “conditioning” regimen is given prior to the infusion of blood or bone marrow-derived stem cells. These regimens serve to make space in the patient’s bone marrow cavity for the incoming donor cells, weaken the patient’s immune system to allow for the acceptance of the donor graft and, in the case of malignant disorders, provide additional anti-cancer therapy. The combination of chemotherapy and/or radiation therapy often results in the development of nausea, vomiting, diarrhea, mucositis, and decreased appetite in transplant recipients [11,15]. The incidence and severity of these symptoms directly correlate with the conditioning regimen’s intensity. Regardless of whether the conditioning intensity is high or reduced, the effects on the patient are significant, setting the stage for malnutrition to be a common finding in pediatric BMT patients [5]; the rates of malnutrition range from 1–47% prior to and 19–20% after BMT [3,16,17]. Importantly, unaddressed malnutrition in this context can lead to significant consequences, including poor growth, development, immune dysregulation, increased hospital length of stay, and increased healthcare costs [2,18,19,20,21].

How malnutrition is addressed varies by BMT center. Generally, two options are considered, with either nutrition (EN) or parenteral nutrition (PN) being introduced. Historically, PN, which is intravenous nutritional supplementation, has been more frequently utilized to address poor nutritional status when oral intake declines during BMT [22]. However, the use of PN has been associated with increases in infection rates, liver dysfunction, and electrolyte imbalances [4,23]. Prior studies have shown the feasibility of enteral nutrition usage in pediatric BMT [24]. The absence of EN, thus bypassing the gastrointestinal (GI) tract, can lead to a loss of microbial biodiversity in the gut [25,26], leading to increased infection rates. The American Society for Parenteral and Enteral Nutrition (ASPEN) recommends the use of EN as the first-line approach over PN and advises PN use only for cases associated with a medical contraindication to EN, including peritonitis, bowel obstruction, or severe GI symptoms [27,28,29]. When patients are unable to orally meet the EN goals, the placement of a temporary feeding tube such as a nasogastric (NG-tube) or gastrostomy (G-tube) is recommended [28,30,31,32].

Many pediatric patients requiring prolonged hospitalization for BMT experience malnutrition, which has been shown to contribute to morbidity, mortality, and prolonged length of stay, and there is a lack of standard-of-care measures to address this problem. Improvement in the nutritional status of these patients can potentially lead to reduced infection and GVHD rates, along with decreased length of stay and healthcare costs. This integrative review aims to evaluate the optimal nutritional support approach for children, adolescents, and young adults (AYA) during BMT admissions.

## 2. Methods

A literature search was performed in January 2024 to evaluate the approaches to and impacts of nutritional support on pediatric and AYA patients undergoing BMT, using the databases of PubMed, CINAHL, Embase, and Cochrane. To narrow the results, the MeSH terms “bone marrow transplantation” or “hematopoietic stem cell transplantation” were combined with the MeSH terms “malnutrition”, “nutrition”, “nutritional status”, or “malnourished.” The results of the first and second search strategies were then combined with a search for the MeSH terms “pediatrics” OR “infant” OR “child” OR “teen” OR “adolescent”. Related keywords in titles and abstracts and truncations of search terms were included in the search strategies. Filters were applied to limit the results to those published within the last 5 years (since 1 January 2019). A total of 720 articles were discovered from the initial search after the removal of duplicates.

An integrative review was conducted according to the Preferred Reporting Items for Systematic Reviews and Meta-Analysis (PRISMA) guidelines [33]. Integrative reviews, as defined within the Johns Hopkins Evidence-Based Practice (JHEBP) Model for Nurses and Healthcare Providers, utilize a mixture of theoretical and research-based literature of various methodologies to evaluate, compare, and summarize, in order to determine if there are gaps within the reviewed literature [34].

**Figure 1:**
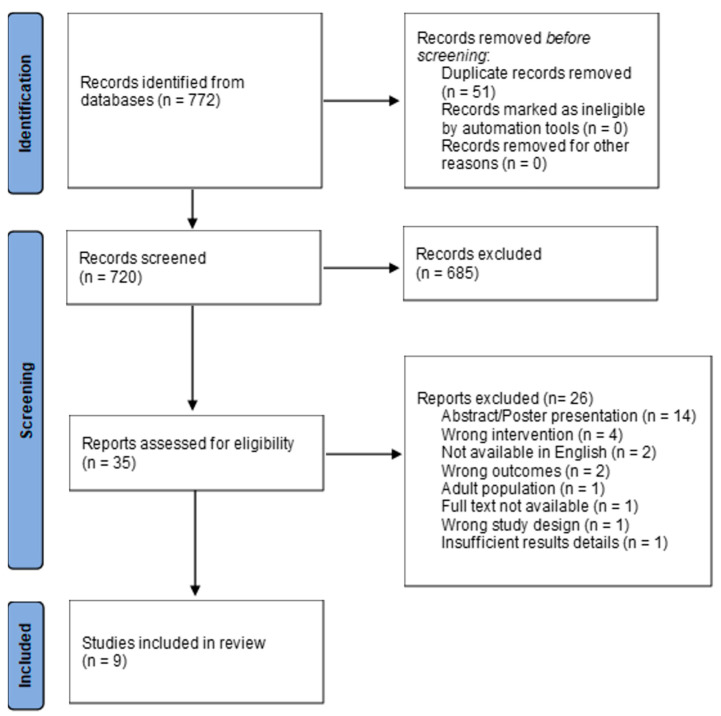
PRISMA flow diagram. Adapted from Ref. [33].

Covidence^TM^ software (https://www.covidence.org/, last accessed on 15 March 2024) was utilized for the synthesis of the literature. Inclusion criteria consisted of studies involving the care of pediatric BMT patients, defined for this review as subjects 20 years of age or under; while the upper age limit for each pediatric cancer center may vary, age 20 years is the cutoff for the CDC pediatric Clinical Growth Charts [35]. Additional inclusion criteria encompassed studies including peri-transplant cases, ideally in an inpatient setting, with a focus on nutritional status and/or malnutrition, and inclusion of a nutritional intervention, such as the use of EN or PN. Articles were excluded if (1) the report did not specifically include BMT patients, focused solely on the pre-transplant period, or involved adult subjects aged 21 or older; (2) the study designs did not contain nutritional interventions; (3) the measured outcomes did not answer the PICO question; or (4) the articles were not available in English. Studies that were not peer-reviewed, such as poster presentations and conference abstracts, or those that included insufficient details, such as a brief report or letter to the editor, were also excluded. After the initial screening, all but thirty-five articles were excluded, and these underwent a full-text review, leaving nine articles that met the inclusion criteria outlined above. See the PRISMA flow chart (Figure 1) for further details. 

After the selection of articles, the JHEBP Research Evidence Appraisal Tool [34] was utilized to determine the quality and strength of the selections. Briefly, per the JHEBP model, the evidence is ranked from level I through level V. Level I, the highest, includes randomized controlled trials (RCTs) or systematic reviews of RCTs. Level II includes quasi-experimental studies, which are without random assignment and/or controls. Level III includes nonexperimental studies, such as retrospective cohort studies. Level IV includes clinical practice guidelines, and level V includes experiential or non-research evidence. The quality of evidence is then graded from A to C: A indicates high quality research with generalizable results, an adequate sample size, the presence of a control, and up-to-date references; B indicates good quality research, with a sufficient sample size, some control, and a degree of up-to-date references; and C indicates low quality research, with inconsistent results, a poor sample size, and no definitive conclusions [34]. 

## 3. Results

Nine articles published between 2019 and 2023 were included for review, as summarized in Table 1. Per the JHEBP guidelines, one study was deemed quasi-experimental and rated Level II, while the remainder were non-experimental and rated Level III. Most studies were deemed to be good quality and rated B, with one rated C. Lack of a control group, small sample sizes, and outdated references were the major contributors to these ratings. The study designs were either prospective, retrospective, or both, and all but one utilized cohorts for convenience sampling. All studies evaluated children following admission for BMT, at centers within Europe, Asia, and Australia. Both allogeneic and autologous BMT recipients were included. The intensity of the transplant regimens varied between reduced- and full-intensity (myeloablation), and the transplant sources included peripheral blood stem cells, bone marrow, and cord blood. The studies included groups of PN only [36], EN with PN [13,37,38,39,40], G-tube vs. NG-tube, or no feeding tube support [37,38,41], analyzing various nutritional and post-transplant parameters. Some patients received prophylactic G-tubes [37,38,41], others had NG-tubes placed by day + 1 [13,24,40,41], and others primarily relied on PN when the oral caloric intake was insufficient [36,39,42]. An alteration in nutritional support regimens was found to have an impact on the ability to meet energy requirements, as well as on various post-transplant outcomes, including length of stay (LOS), engraftment, overall survival (OS), and the incidence of acute GVHD (aGVHD), infection, and veno-occlusive disease (VOD).

### 3.1. Intervention Threshold

The studies varied with respect to when nutritional support was introduced, as well as the means to measure the ongoing nutritional status of the patients. Various ways to measure the overall nutritional status included food weighing and recording daily intake [37], daily weights [13,36,37,38,39,43], and the albumin level [13,36,37]. EN was typically started when patients were unable to meet more than 50% of their caloric needs over 2–3 days; however, some calculated this by the Holliday–Segar formula [41] and others by the Scientific Advisory Committee on Nutrition’s recommendations [37]. Approaches to EN varied between boluses, continuous for 12–20 h, or for 9–12 h overnight to supplement daytime PO intake [36,37,41]. PN was added in cases of severe mucositis, GI GVHD, NG-tube refusal, or intractable vomiting and/or diarrhea [13,36,37,41], or otherwise in cases of an inability to meet greater than 50% of their caloric needs by EN support [37]. In studies that utilized prophylactic G-tube placement, one study found that, on average, the surgery occurred around 46 days prior to BMT admission [43], but in others, this timing was not noted [37,38,39,41]. When noted, NG-tubes were placed from day − 2 to day + 2 [40]. Several studies did not explain the measures used to evaluate nutritional status or detail the specific indications for utilizing EN or PN support [13,36,38,39,42,43].

### 3.2. Energy Requirements

The inability to meet at least 75% of the estimated calorie/protein needs is a marker of malnutrition [27]. In the Lewandowki et al. [39] study, none of the patients were able to meet 75% of their needs; however, the oral intake plus supplemental EN group was the most successful, followed by the oral intake with PN group. One study utilized a nutritional support algorithm with universal NG-tube placement and supplemental EN, started when oral intake fell below 75%, for 3–5 days. PN was subsequently added for EN intolerance, with continued trophic feeds when possible [13]. The 2023 Evans study also utilized a combination of oral intake, as noted by a daily food diary, EN calculation, and PN calculation, to ensure all patients met the recommended calorie and nutrient goals [41]. The adequacy of nutrition support in relation to caloric intake or overall dietary intake was not discussed in other studies [36,38,40,42].

### 3.3. Length of Stay (LOS)

LOS was evaluated in five studies. Patients who exclusively received supplemental EN were found to have a decreased LOS compared to those who received EN with PN or PN only, in one study [13]. When comparing the prophylactic placement of G-tubes prior to BMT with standard NG-tube placement, there was no difference in LOS between groups nor between the EN only and EN-PN groups [37]. There was also no difference in LOS based on overall nutritional status in the Lewandowski et al. [39] study. Unfortunately, there was no differentiation of LOS based on the types of nutritional support received.

### 3.4. Engraftment

The time to platelet or neutrophil engraftment was evaluated in three studies. In one study, earlier platelet engraftment was seen in the group receiving supplemental PN to a count of >20 × 10^9^/L, but this difference was not significant at a threshold of >50 × 10^9^/L [40], whereas another study found no impact of supplemental nutrition on count recovery [38]. Although neutrophil engraftment occurred earlier in a group of patients with prophylactically placed G-tubes versus those with NG-tubes in one study [43], two other studies did not find a difference [38,40].

### 3.5. Acute Gastrointestinal (GI) GVHD

Acute gastrointestinal (GI) GVHD was found to be less frequent when supplemental EN was employed as much as possible over PN use, in one study [42]. By contrast, there were no statistically significant differences in aGVHD between the EN group and the PN groups in the Zama et al. [40] nor between the G-tube and NG-tube or non-feeding tube groups in other studies [37,38,41,43]. GVHD rates as high as 33% in patients were found in a study that primarily utilized PN support when optimal oral intake could not be achieved, but no association was made between the nutritional support type and the GVHD diagnosis [39]. The diagnosis of acute GI GVHD was associated with the need to start PN later in the post-BMT course in one study involving primary immunodeficiency patients [13].

### 3.6. Infections

Rates of infections were quantified in all but one study, and they were compared between EN and PN nutritional support groups in two studies. The Zama et al. [40] paper found the rate of bloodstream infections decreased in the group of patients receiving supplemental EN compared to the PN group. In a study that evaluated only patients who received PN, a significant number of infections were found to occur in pediatric BMT patients, and the rates of complications correlated with the length of PN use [36]. However, no significant differences in bloodstream infection or overall infection rates were noted between the nutritional support groups in three other studies [37,41,42]. Mellgren et al. observed increased febrile episodes attributed to G-tubes within the first three months post-BMT when compared to patients with NG-tubes; however, no infections were life-threatening [43]. Another study found that 89% of patients had at least one infection during their BMT admission. While 45% of patients received PN, the rates of infection between those who received PN versus those who did not were not evaluated [39]. Finally, rates of infection of 15–19% were observed in pediatric immunodeficiency patients undergoing BMT; however, the association with the methods of nutritional support was not evaluated [13].

### 3.7. Veno-Occlusive Disease (VOD)

Severe veno-occlusive disease (VOD), also known as sinusoidal obstruction syndrome (SOS), can be a life-threatening complication following BMT. The incidence of VOD was evaluated in the context of nutritional support in two studies, with contradictory results. In the Alsalamah et al.’s [42] study, the incidence of VOD was found to be significantly higher in the group of patients receiving supplemental PN; however, the majority of these patients received myeloablative conditioning and were otherwise at increased risk for the development of VOD due to elevated ferritin levels pre-BMT. No differences were found regarding the incidence of VOD between recipients of supplemental EN and PN in the Zama et al.’s study [40].

### 3.8. Overall Survival

Overall survival (OS) was evaluated in three studies, with mixed results. The OS was higher and the risk of leukemia relapse lower in patients receiving G-tube nutrition compared to those who did not receive any tube feedings, in one study [38], while others did not show a significant difference in OS between groups [37,43].

## 4. Discussion

This integrative review highlights what is currently known about nutritional support for children undergoing BMT. Practice approaches to the initiation of EN versus PN varied greatly among studies, with no consensus reached, and the impact on patient outcomes is unclear. From the literature, it is evident that EN use, including prophylactic placement of G-tubes, is feasible and not inferior to the utilization of PN or NG-tubes. However, there was no consensus regarding the appropriate timeline of placing these tubes. Although inconclusive from this review, there are some data to suggest that supplemental EN use may contribute to reduced infection rates, as well as a decreased incidence and severity of GVHD [36,40,44].

ASPEN has advocated for the use of supplemental EN as the first line for nutritional support for all children with malnutrition [28], and this is echoed by the European Society for Parenteral and Enteral Nutrition (ESPEN) [45], as well as the European Society for Blood and Marrow Transplantation (EBMT) [46,47]. Unfortunately, the implementation of this approach is not being executed uniformly in the pediatric BMT population, despite some evidence of its benefits, including decreased length of stay and lower costs [22,48]. In a multicenter, randomized, controlled trial comparing EN versus PN use in critically-ill adults, minimal differences in outcomes were seen between groups, except for higher costs with PN [49]. By contrast, EN use in combination with PN contributed to improved patient outcomes in other studies, highlighting the potential benefits of continuing EN at any volume possible to maintain a diverse gut microbiota [22,50,51]. NG tube placement is usually performed by day + 1 [43,52]; however, in centers using post-transplant cyclophosphamide, this may be pushed to day + 5 [53]. Some studies turned to prophylactic surgical G-tube placement to help increase the utilization of EN [38,41,43], while others only administered PN during the nighttime, resulting in increased EN intake during the daytime [54]. Barriers to the routine use of EN are multi-factorial and include patient-related, institutional, and pragmatic issues, such as the presence of mucositis or GI distress, provider and/or family perceptions and biases, lack of institutional or national clinical guidelines, and a paucity of randomized-controlled trials, contributing to a lack of high-quality evidence to support practice changes [22].

Malnutrition in children with cancer and those undergoing BMT has been found to be correlated with decreased survival [44,54]. Loeffen et al. evaluated malnourished pediatric oncology patients receiving initial chemotherapy and found significantly decreased survival rates among children who were malnourished at diagnosis and 3 months after diagnosed, compared to adequately nourished children [55]. GVHD is a significant cause of non-relapse-related mortality in BMT patients; therefore, examining ways to decrease the incidence of GVHD is of utmost importance. Kerby et al. [51] found that a diagnosis of malnutrition in the previous 30-day period was associated with a three-to-fourfold increased risk of developing severe aGVHD, and this was hypothesized to be related to the pro-inflammatory nature of malnutrition [54]. The gut microbiome is also understood to play a role in patient outcomes; lack of a stimulated and diverse microbiome, such as that observed with the exclusive use of PN, could contribute to increased rates of GVHD and infections [22,44,50]. Vitamin D is also thought to play a role in immunoregulation and gut inflammation, with several adult and pediatric studies showing increased rates of acute and chronic GVHD in patients with documented vitamin D deficiency [56,57].

Similar findings can be seen in reviews of adult literature regarding malnutrition and nutritional support during BMT [58,59]. In a trial of adults who underwent autologous BMT, a nutrition optimization protocol that featured early initiation of EN and targeted PN was associated with a decreased rate of infections, as well as shorter LOS [58]. There was no difference in time to platelet engraftment, and overall survival was not measured past day +30 [58]. Hirose et al. [59] found that severe malnutrition in adult patients prior to the start of allogeneic BMT was associated with a higher risk of acute GVHD, increased non-relapse mortality, and decreased OS, illustrating that there is a role for optimization of nutritional status, even before the patient is admitted for BMT. The EBMT has published guidelines for improving nutrition in BMT patients. However, these are largely extrapolated by studies in adults, and the authors note that specific pediatric guidelines are lacking [46]. Key points from these recommendations include malnutrition risk screening prior to and frequently during BMT, EN usage over PN, the elimination of the usage of low-microbial or neutropenic diets, and prophylactic NG-tube placement on day + 1 [46]

Equally important, this review reveals several rather glaring knowledge gaps regarding the topic of nutritional optimization in children during BMT, thus exposing a significant unmet need in the field. The majority of studies reviewed attempted as much EN support as possible, but ultimately many also required PN use, so it remains unclear whether EN or PN is the superior approach for nutritional optimization, and likely some combination of both will always be required for BMT patients. It would be beneficial to further clarify the impact of nutritional optimization on outcomes post-BMT, determine whether these impacts are significant, and if these effects occur in allogeneic BMTs, autologous, or both. Particular outcomes that might be improved or studied, such as GVHD, infection, length of stay, and overall survival, should be analyzed with respect to the malnutrition status and optimization. A meta-analysis comparing EN versus PN support after BMT, performed by Zama et al. in 2021, showed that the primary use of EN reduced high grade GI aGVHD; however, this analysis primarily contained adult data and included studies that were over 20 years old [60]. Malnutrition screening should be assessed early and often for pediatric BMT patients. However, clear guidelines for when supplemental nutrition should be started, whether prophylactic NG- or G-tubes are warranted, and how to determine the duration of need for these tubes, are lacking. Creating standardized pediatric BMT nutritional support guidelines is not possible until further studies are performed.

This review does contain limitations. Firstly, the lack of research from North American centers limits global generalizability. A limited number of articles met the inclusion criteria, and those included contained small sample sizes, limiting applicability. There were no randomized controlled trials available to be included in this review, and most studies utilized cohort sampling, allowing for sampling errors. The majority of studies were retrospective, allowing for recall bias, as well as errors due to loss of documentation or misinterpretation over time. Most of the articles were of good quality (B); however, none of the articles included were of high quality (Quality A). In some cases, outcomes varied between studies utilizing similar measurements, which makes the interpretation of results particularly challenging. EN tolerance was associated with less intense conditioning regimens in some studies, making it difficult to evaluate if the improved outcomes were due to the less severe regimen-related toxicity or the nutrition support itself. In the studies that evaluated GVHD, there was no discussion on the use of GVHD prophylaxis, and varying donor types and stem cell sources were utilized, further confounding the results. Using both autologous and allogeneic BMT platforms, along with variations in the intensity of preparative regimens and stem cell sources, further hinders the ability to apply these findings across the general pediatric BMT population.

## 5. Conclusions

Unaddressed malnutrition can significantly impact morbidity and mortality for children who are undergoing BMT. Survival rates for childhood cancer and BMT continue to increase, but there is much room for improvement. Complications during BMT, including infections, GVHD, the need for frequent platelet transfusion requirements, and increased healthcare-associated costs due to prolonged hospitalizations, can potentially be decreased via the optimization of EN. Many children are unable to meet caloric needs orally during their BMT admission, and there is no current consensus on how to best address this. Each pediatric BMT center needs to develop practice guidelines to better serve these patients. The paucity of published literature on this topic limits the conclusions that can be drawn from this review and illuminates a critical unmet need in the field. Future research should focus on ways to improve the early initiation of nutritional support, including determining the best timeline for the initiation of supplemental nutrition, as well as increasing enteral feeding tube placement and utilization to optimize EN administration in children who are admitted for BMT. Reaching a consensus on how to best measure the nutritional status of a pediatric BMT patient, as well as what outcomes to measure following nutritional interventions, will be important for providing more conclusive recommendations. Prospective studies will be useful in determining the most effective interventions to support these nutritional goals.

## Figures and Tables

**Table 1:** Table of evidence.

Author and Date	Evidence Type	Sample, Sample Size, Setting	Intervention	Findings That Help Answer the EBP Question	Observable Measures	Limitations	Evidence Level, Quality
Alsalamah et al., 2022 [42]	Retrospective cohort study	Children <14 undergoing BMT at a hospital in Saudi Arabia PN (*n* = 144) EN (*n* = 84)	Review of nutritional support (PN, NG-tube, oral) use and complications	PN used most over NG and oral, associated with increased VOD, aGVHD, and mucositis. Reduced-intensity conditioning patients less likely to use TPN by 70%	Nutritional support length of time by type, incidence of mucositis, VOD, aGVHD, infection. Measurement interval periods not defined.	Retrospective design. Small sample size. Unclear if PN groups also had EN.	Level III, Quality B
Evans et al., 2023 [41]	Prospective cohort study	Children 1–9 undergoing allogeneic BMT at an English hospital G-tube (*n* = 24) NG-tube (*n* = 19)	Prophylactic G-tube placement vs NG-tube. Included some PN use	Both groups experienced at least 1 complication, majority minor. No statistically significant differences between groups on measured outcomes.	Nutritional support means and length of time, incidence of tube complications, infections, weight, height, MUAC, caloric and nutrient intake, OS, non-relapse mortality, GVHD. Measured weekly from Day −7 to day +42, then monthly until day +180.	Small sample size. G-tubes primarily in non-malignancies. PN added if unable to meet energy caloric requirements (first-line PN use excluded) but usage of PN not reported.	Level III, Quality B
Evans et al., 2019 [37]	Retrospective cohort study	Children <18 undergoing BMT after reduced-intensity or myeloablative conditioning at an English hospital G-tube (*n* = 54) Non-G-tube (*n* = 91) Subgroups: EN only (*n* = 34) EN-PN (*n* = 96)	Prophylactically placed g-tube vs without (PN, NG-tube, oral, and combinations) with G-tube subgroup of EN only vs EN with PN	Decreased length of stay in EN only subgroup compared to EN-PN. No difference in LOS, OS at day 100, incidence of aGVHD, infection rates between g-tube and no G-tube and EN alone vs EN-PN	Changes in nutritional status, and post-transplantation outcomes. Measurement interval periods not defined, but included admission, discharge, and Day +100	Absence of randomization and control group. Retrospective design with missing data. Excluded cord blood patients. Large number of immunodeficiency patients.	Level III, Quality B
Kairiene et al., 2023 [38]	Retrospective cohort study	Children <18 admitted for allogeneic BMT at Lithuanian hospital G-tube (*n* = 34) Non-G-tube (*n* = 41)	Prophylactically placed g-tube vs without feeding tubes. Included some PN	Improved transplant-related mortality and 5-yr OS with PEG	MRD status, time of G-tube use, changes in nutritional status, caloric intake, time to engraftment, aGVHD, 5-year event-free survival, OS.	Retrospective over 12 yrs.; practice change during that time. PN added if unable to meet energy caloric requirements; usage not reported. More marrow vs peripheral blood in G-tube group. Some loss of data.	Level II, Quality B
Lewandowski et al., 2019 [39]	Retrospective cohort study	Children 0–19 admitted for BMT in a Brazilian hospital (*n* = 63)	Review of nutritional status, support used, and complications	No variation in LOS among nutritional diagnoses. Higher rate of PN than EN in all patients. A total of 89% of patients had at least one infection, 21% had GVHD.	Nutritional support means, LOS, time to engraftment, incidence of GVHD, GI symptoms, infection rate, caloric intake, weight gain/loss. Measured at admission, day 0, +14, +21, and +28	Not originally published in English, possible translation errors. BMT-related outcomes were not delineated by type of nutritional support received. No control.	Level III, Quality C
Mellgren et al., 2023 [43]	Retrospective cohort study	Children <18 yrs. admitted for allogeneic BMT at three Swedish hospitals G-tube (*n* = 112) NG-tube (*n* = 115)	Prophylactically placed G-tube vs NG-tube, Included some PN	NG-tube group lost more weight. G-tube group had earlier neutrophil engraftment but more fevers/infections. No differences in aGVHD, OS, non-relapse mortality, relapse.	Nutritional support means and time usage, non-relapse mortality, relapse, incidence of GVHD, infection, time to engraftment, height, weight. Measured at time of BMT, +90, +180, and +365	Multi-center with 1 center with universal G-tube placement prophylactically. A total of 43 patients deceased or lost to follow-up. PN use not equal between groups.	Level III, Quality B
Soussi et al., 2019 [36]	Retrospective cohort study	Children 2–17 undergoing allogeneic BMT at a hospital in Tunisia, receiving PN (*n* = 51)	Review of PN use and complications	Total of 136 observed complications. Infections were the most common complications (32%), then electrolyte disorders (28%). Risk correlated with duration of PN exposure. GVHD in 29%.	Incidence of infections, electrolytes/metabolic imbalances, hepatobiliary complications, mucositis, GVHD, death. Measured from date of PN initiation to completion	Overestimation of PN complications due to difficulty to confirm association of PN use with cause. Reports difficulty with collecting biological parameters reliably. No control.	Level III, Quality C
Zama et al., 2020 [40]	Retrospective and prospective cohort study	Children 2–20 undergoing myeloablative or non-myeloablative conditioning for allogeneic BMT admitted to an Italian hospital PN group (*n* = 28) EN-PN group (*n* = 14)	Comparison between group receiving EN at least 7 days vs. primarily PN	The rate of bloodstream infections was higher in PN group. Trend towards lower rate of severe and steroid-resistant aGVHD in EN group. Longer time to platelet recovery in EN group. No difference in neutrophil engraftment, days of GCSF, LOS, mucositis, aGVHD.	Nutritional status, weight loss, and clinical outcomes including neutrophil and platelet engraftment, mucositis, aGVHD, VOD, and blood stream infection occurrences. Measured on admission, weekly until Day +35, discharge, +60, and +90	Mix of patients who had only PN and those who received PN with EN for up to six days in one group, and those who received PN with EN for more than seven days or EN only in 2nd group. Small sample size.	Level III, Quality B
Zemrani et al., 2019 [13]	Retrospective cohort study	Children <20 with primary immunodeficiencies (PID) undergoing reduced-intensity or myeloablative conditioning allogeneic BMT at an Australian hospital (*n* = 27; 31 BMTs)	Review of nutritional status, support used, and complications	A total of 33% of patients met criteria for malnutrition prior to BMT. EN was initially used for most. A total of 77% required PN. Exclusive EN was associated with shorter LOS. PN associated with hyperglycemia and hyper-triglyceridemia.	Nutritional support means, weight, height, albumin/electrolytes, neutrophil and platelet engraftment date, mucositis, acute and chronic GVHD, infection rate, VOD, LOS, OS. Measured at start, Day +30, +90, +180, and +365	BMT-related outcome measures were not delineated by nutritional support received or prep intensity. No control/comparison group. Results are not generalizable to non-PID BMT patients.	

Note: BMT = blood and marrow transplantation; EN = enteral nutrition; PN = parenteral nutrition; LOS = length of stay; OS = overall survival; GVHD = graft-versus-host disease; aGVHD = acute graft-versus-host disease; VOD = veno-occlusive disease; G-tube = gastrostomy tube; NG-tube = nasogastric tube; MUAC = mid-upper arm circumference; GCSF = granulocyte colony-stimulating factor.
